# Securin promotes the identification of favourable outcome in invasive breast cancer

**DOI:** 10.1038/sj.bjc.6605237

**Published:** 2009-08-18

**Authors:** K Talvinen, H Karra, S Hurme, M Nykänen, A Nieminen, J Anttinen, T Kuopio, P Kronqvist

**Affiliations:** 1 Department of Pathology, University of Turku, Kiinamyllynkatu 10, Turku FIN-20520, Finland; 2 Department of Biostatistics, University of Turku, Lemminkäisenkatu 1, University of Turku, Turku FI-20014, Finland; 3 Department of Pathology, Jyväskylä Central Hospital, Keskussairaalantie 19, Jyväskylä FIN-40620, Finland

**Keywords:** securin, breast cancer, prognosis, proliferation

## Abstract

**Background:**

Securin is a recently recognised oncogene with multiple known functions in initiation, progression and cell cycle regulation in several malignant diseases, including breast carcinoma.

**Methods:**

In this paper, the prognostic value of securin is evaluated by immunohistochemistry in 310 patients diagnosed with invasive breast cancer during a mammographic screening programme in Central Finland. All patients were directed to modern surgical and oncological treatments and were followed up for a maximum of 20 years.

**Results:**

Our results suggest that securin immunopositivity is an independent prognosticator of invasive breast cancer. In our study, securin predicted breast cancer-specific survival among all cases of invasive breast cancer and subgroups divided according to histological type, Ki-67 proliferation status and tumour size. Especially in a multivariate analysis standardised for axillary lymph node status, patient's age and tumour size at the time of diagnosis, securin immunopositivity indicated a 13.1-fold risk of breast cancer death (*P*=0.024) among invasive ductal breast carcinomas with low Ki-67 positivity.

**Conclusion:**

Our present and previous results suggest that securin could be useful in clinical pathology to intensify the power of the established prognosticators of invasive breast cancer and, especially, to assist in identifying patients with a more favourable outcome than that indicated by Ki-67 alone.

Despite advances in diagnostics, treatment and overall prognosis of breast cancer, the outcome of individual breast cancer patients may still be unpredictable and relapses occur after several years from primary treatment (Thompson *et al*, 2008). In recent years, features underlying the heterogeneous behaviour of breast cancer have been addressed with the help of modern methods of molecular pathology and histopathology (van de Vijver *et al*, 2002; Rakha *et al*, 2008; Geyer *et al*, 2009). In several investigations, uncontrolled mitotic activity has been recognised as being among the most important factors influencing breast cancer development and progression (Stuart-Harris *et al*, 2008; Baak *et al*, 2009). Uncontrolled aberrant mitotic divisions leading to aneuploidy have been suggested in literature as being one of the most common characteristics of any cancer cell and also with a strong influence on the behaviour of breast cancer (Bharadwaj and Yu, 2004; Kops *et al*, 2005). This paper reports on the role of securin (pituitary tumour transforming gene, PTTG1) in estimating proliferative activity and prognosis in breast cancer. Securin is an oncogene with a known role in cell cycle regulation and maintenance of chromosomal integrity (Tfelt-Hansen *et al*, 2006; Bradshaw and Kakar, 2007; Salehi *et al*, 2008).

Securin was first isolated in rat pituitary tumour cells (Domínguez *et al*, 1998). Thereafter, securin has been identified in human tissues with a low expression in normal cells but elevated expressions in proliferating cells of several malignant tumours. To date, securin has been reported to be overexpressed, especially in endocrine-related tumours such as thyroid, breast, ovarian and uterine carcinomas, and also in non-endocrine tumours such as pulmonary and gastrointestinal carcinomas and malignancies of the central nervous system (Shibata *et al*, 2002; Solbach *et al*, 2004; Wen *et al*, 2004; Ogbagabriel *et al*, 2005; Salehi *et al*, 2008). Securin is a multidomain and multifunctional protein identified with roles in cell cycle, cellular transformation, tumour development and angiogenesis, although the exact molecular mechanisms of these interactions are for the most part still unrevealed (Salehi *et al*, 2008). Securin has particularly been suggested to participate in the regulation of chromatid separation during the metaphase–anaphase interface of the cell cycle and to thus take part in maintaining chromosomal stability (Tfelt-Hansen *et al*, 2006; Bradshaw and Kakar, 2007; Salehi *et al*, 2008). According to clinical studies, securin overexpression associates with the spread, invasion and metastasis of cancer cells and predicts the outcome of several malignancies (Boelaert *et al*, 2003; Ramaswamy *et al*, 2003; Ito *et al*, 2008). In addition, overexpression of securin has been related to endocrine therapy resistance in breast cancer (Ghayad *et al*, 2009).

Securin has also been reported to be overexpressed and associated with disease outcome in invasive breast carcinomas (Sáez *et al*, 1999; Solbach *et al*, 2004; Ogbagabriel *et al*, 2005). Our previous paper implied the prognostic value of securin on the basis of a cDNA gene expression analysis, and immunohistochemical analyses further suggested that securin might intensify the prognostication of patients with aggressive disease (Talvinen *et al*, 2008). This paper extends the prognostic value of securin immunohistochemistry in invasive breast cancer on the basis of patient material (*n*=310) obtained during a mammographic screening programme and followed up for a maximum of 20 years.

## Materials and methods

### Patient data

The study comprises a total of 310 unselected patients diagnosed with invasive breast cancer and treated in the Jyväskylä Central Hospital, Finland, during 1987–1997 when mammographic screening was systematically practised in Central Finland. Treatment of all patients included resection or mastectomy with axillary evacuation, and adjuvant treatment with anti-oestrogen and/or cytostatic drugs depending on the patient's age, hormone receptor status and axillary lymph node status at the time of diagnosis. No pre-operative adjuvant treatment was administered. Patients were followed up for an average of 10.5 post-operative years. Causes of death were based on autopsy reports, death certificates and patient files from the Finnish Cancer Registry. Complete clinical and follow-up data are summarised in Table 1.

### Tissue microarrays

The histological material of each breast cancer patient were arranged in tissue microarrays (TMAs). The representative breast cancer area from routinely formalin-fixed and paraffin-embedded breast cancer blocks from archive material was identified. Two tissue cores (diameter 0.6 mm, min height 5 mm), one from the centre and the other from the edge of the representative breast cancer area of each paraffin block, were punched and precisely arranged into three TMA blocks with 238–258 cores in each, as presented by Kononen *et al* (1998).

### Immunohistochemical methods

Immunostainings of securin and Ki-67 were applied on 3 *μ*m formalin-fixed and paraffin-embedded TMA sections according to the standard procedure using the automated immunostaining machine, LabVision Autostainer 480 (Thermo Fisher Scientific, Fremont, CA, USA). Antigen retrieval was performed in a microwave oven at 99°C for 10 (securin) or 15 (Ki-67) min in a citrate buffer (Dako REAL Target Retrieval Solution, Dako, Glostrup, Denmark, S2031, 1 : 10). Antibodies were applied at concentrations of 1 : 50 (securin, clone DCS-280, ab3305, Abcam, Cambridge, UK) and 1 : 100 (Ki-67, 18-0192, Zymed, Invitrogen, Carlsbad, CA, USA). Detection was performed using a PowerVision+ polymer Kit according to standard protocols (DPVB+110HRP, ImmunoVision Technologies, Vision BioSytems, Norwell, MA, USA) with diaminobenzidine as chromogen. All malignant cells of each tissue cylinder were counted. Positively stained securin and Ki-67 nuclei were registered as a fraction (%) of the number of tumour cells. In our previous paper, we presented intraclass correlation coefficients for intra- and interobserver reproducibilities of securin and Ki-67 immunoevaluations (ICC 0.51–0.96) (Talvinen *et al*, 2008).

### Statistical analysis

To explore the prognostic value of securin and Ki-67, we elaborated a threshold based on follow-up information of the patient material by screening with univariate analyses and selecting points of strongest prognostic value. Selected cut-off points were confirmed by receiver operating characteristic analysis. To evaluate the potential of securin to intensify the value of Ki-67 in breast cancer prognostication, we analysed all possible combinations of low and high expressions of both securin and Ki-67 immunopositivity by forming four classes of patients: low securin and low Ki-67, high securin and low Ki-67, low securin and high Ki-67, and high securin and high Ki-67.

The present analysis allowed us to estimate the consistency related to the histological TMA method by comparing results from tissue punches representing different areas of the specimen, one punch from the centre and another from the periphery of the tumour. Comparison of immunopositivity of the central and peripheral tumour areas showed moderate consistency (ICC 0.737 for securin and 0.887 for Ki-67). Moreover, McNemar's test of marginal homogeneity (*P*=0.63 and *P*=0.45 for securin and Ki-67, respectively) and kappa coefficients (*κ*=0.59 and *κ*=0.69 for securin and Ki-67, respectively) showed concordance between central and peripheral tissue cores. The observed acceptable consistencies between the results of central and peripheral tumour areas allowed us to apply for final prognostic analyses either the mean of the results of the two tissue cores (65% of cases) or a single observation. A single observation was used if only one tissue core was representative of cancer cells.

Survival analysis was performed to investigate the prognostic value of securin and the association between securin and Ki-67 in evaluating breast cancer outcome. The cumulative percentages for breast cancer-specific mortality were estimated using the Kaplan–Meier technique and the differences between categorised values were tested using the log-rank test. Differences between categories were quantified by calculating hazard ratios (HRs) with 95% confidence intervals (95% CIs) using Cox's regression models. Cox's regression models with multiple explanatory variables were used to adjust the results for axillary lymph node status classified as node-positive and node-negative cases, with patient's age and tumour size as continuous variables. An adjusted analysis was made separately for securin, Ki-67 and the variable established by the combination of the two. Patients with missing data were excluded from analyses. *P*-values less than 0.05 were considered to be statistically significant. All statistical computations were performed using SAS System for Windows, Version 9.1.3 and SAS Enterprise Guide 4.1. (SAS Institute Inc., Cary, NC, USA).

## Results

Table 2 summarises the results of securin and Ki-67 immunohistochemistry in 310 cases of invasive breast cancer and in patient subgroups divided according to tumour histology and Ki-67 status. The observed fraction of securin immunopositivity was systematically lower than that of Ki-67 throughout all cases and prognostic subgroups. Screening with univariate survival analyses and selecting points of strongest prognostic value set an optimal cut-off point at 1.5% for securin immunopositivity. This cut-off point produced two patient groups, which, with the highest statistical significance, identified patients who were alive with or who died of breast cancer during the follow-up period. Receiver operating characteristic analysis of securin immunohistochemistry validated the selected cut-off point. The same methods confirmed the established threshold of 10% immunopositivity as the optimal cut-off point for Ki-67 in our study (Tavassoli and Devilee, 2003, p 19).

**Figure 1:**
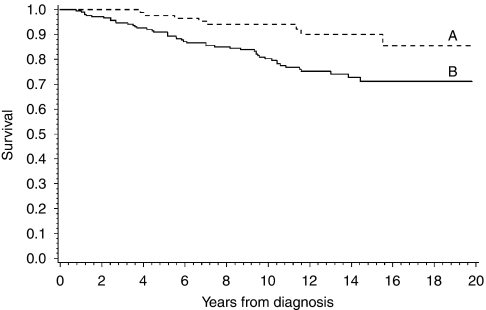
Kaplan–Meier curves of breast cancer survival based on securin immunohistochemistry in 310 breast cancer patients. Securin immunohistochemistry (positivity <1.5% (A) *vs* ⩾1.5% (B) of cancer cell nuclei) distinguishes patients with a different outcome of disease (*P*=0.004).

Figure 1 shows the potential of securin to separate the patients into groups of favourable and unfavourable outcome of breast cancer on the basis of a cut-off point of 1.5% immunopositivity (*P*=0.004). Similarly, the prognostic significance of securin was shown in subgroups of invasive ductal histology (*P*=0.010), and with small and large tumour size (*P*=0.034 and *P*=0.033 for tumour diameters ⩽3 cm and >3 cm, respectively). The results of Cox's univariate survival analyses are interpreted as a comparison of the survival between groups of patients associated with securin and Ki-67 immunopositivity above and below the determined cut-off points at 1.5 and 10%, respectively. On the basis of an analysis of all cases and subgroups of ductal histology of breast cancer, securin predicted the survival of disease with statistical significance (Table 3). In comparison with the prognostic values of securin and Ki-67, univariate Cox's survival analysis for securin indicated a 2.9-fold (*P*=0.006, 95% CI 1.3–6.0) and for Ki-67 a 2.4-fold risk of breast cancer death (*P*=0.004, 95% CI 1.3–4.5). In a combination of securin and Ki-67, the observed risk of breast cancer death was 5.8-fold (securin⩾1.5 and Ki-67⩾10% *vs* securin<1.5 and Ki-67<10%, *P*=0.004, 95% CI 1.8–18.9). In addition, when compared, the high to low securin immunopositivity risk for breast cancer death was 2.7-fold for patients with small and 3.6-fold for patients with large tumour size (*P*=0.042, 95% CI 1.0–7.0 and *P*=0.046, 95% CI 1.0–12.4, respectively).

When the data of 310 breast cancer cases were adjusted for nodal status, patient's age and tumour size, securin immunopositivity still seemed to be a statistically significant predictor for outcome of invasive breast cancer (Table 4). In this analysis, the observed HR of breast cancer death was 2.3 for securin (*P*=0.028, 95% CI 1.1–5.0). As expected, nodal status (*P*=0.0004) and tumour size (*P*<0.0001) were also significant predictors of breast cancer death. The corresponding analysis of Ki-67 indicated a similar prognostic potential, although statistical significance was not quite attained in our study (*P*=0.058, HR 1.9). The highest prognostic value was associated with the combination of securin and Ki-67, which predicted a 4.3-fold risk for breast cancer death in our study (*P*=0.017, 95% CI 1.3–14.2). In addition, in the subgroup having low proliferative activity (Ki-67<10%), securin predicted a 5.1-fold risk of breast cancer death (*P*=0.031, 95% CI 1.2–22.7). Further analysis of patients with low Ki-67 positivity and invasive ductal histology associated securin immunohistochemistry with a 13.1-fold risk of breast cancer death (*P*=0.024, 95% CI 1.4–121.3). Similarly, securin immunopositivity predicted the disease outcome among patients with large tumour size (>3 cm in diameter) when adjusted for patient's age and nodal status (*P*=0.040, HR 3.8, 95% CI 1.1–13.7).

## Discussion

Securin is a recently recognised oncogene with multiple known functions in the tumourigenesis and progression of malignant diseases, especially in the regulation of chromosome integrity in cell proliferation. In breast cancer research, knowledge about securin is fast accumulating, but to date, there are no published prognostic studies on securin in invasive breast cancer patients with long-term follow-up.

Our results suggest that securin immunohistochemistry is an independent prognosticator of invasive breast cancer. This finding is in concordance with our previous paper comprising a small sample size of aggressive breast carcinomas with a high proliferation rate (Talvinen *et al*, 2008). In this paper on 310 breast cancer patients with a long-term follow-up, low securin immunopositivity indicated a favourable course of disease, especially in association with low Ki-67 immunopositivity. In our results, the combination of high immunopositivity for both securin and Ki-67 indicated a 4.3-fold risk of breast cancer death as compared with the prognostic value of low securin and Ki-67, suggesting that securin in combination with Ki-67 enhances the prognostic information derived from cell proliferation. In the light of our results, securin may prove to be a valuable prognostic factor for clinical pathology, although the present data do not yet allow for testing of the possible prognostic value of securin in relation to the gold standard of breast cancer prognostics, the Nottingham Prognostic Index (Elston and Ellis, 1991).

According to literature, securin is involved in the regulation of cell cycle as a mitotic check-point gene, functioning in the metaphase–anaphase transfer (Hagting *et al*, 2002). Securin is critical for genetic stability, as it inhibits premature sister chromatid separation. In detail, during metaphase, cohesin binds sister chromatids and degradation by separase is necessary to enable sister chromatid separation at anaphase (Ciosk *et al*, 1998; Nasmyth, 2005). According to present knowledge, securin is involved in arresting metaphase by binding to separase, preventing cohesin degradation and thus blocking chromatid separation (Waizenegger *et al*, 2002; Nasmyth, 2005). Despite this biological understanding of the function of securin during a normal cell cycle, the role of securin in the proliferation of malignant cells is still unsettled. Some investigations suggest that securin overexpression could reduce cell proliferation by arresting mitosis (Yu *et al*, 2000). Others have speculated that securin could function in cell proliferation through the induction of apoptosis and delay of mitosis (Akino *et al*, 2005). Inhibition of sister chromatid separation, in turn, suggests that securin is responsible for uneven chromatid separation and induction of aneuploidy in tumourigenesis and tumour progression (Tfelt-Hansen *et al*, 2006; Vlotides *et al*, 2007; Salehi *et al*, 2008). Available clinical studies, however, demonstrate a strong correlation between securin expression and cell proliferation in different malignancies (Heaney *et al*, 2002; Tsai *et al*, 2005; Filippella *et al*, 2006; Genkai *et al*, 2006; Zhu *et al*, 2006).

As a proliferation marker, securin clearly differs from established markers of cell proliferation in clinical pathology. The established proliferation markers, Ki-67 and proliferating cell nuclear antigen (PCNA), are expressed in all phases of the cell cycle (Takasaki *et al*, 1981; Guillaud *et al*, 1989; van Oijen *et al*, 1998). In comparison with these, securin concentrates on a specific phase of the cell cycle, the expression gradually increasing during the S phase with a peak at G2/M (Yu *et al*, 2000; Chen *et al*, 2005; Chao and Liu, 2006). According to many investigations, specific fractions of the cell cycle might provide relevant information on the behaviour of different malignancies. Therefore, markers of selected phases of the cell cycle could prove beneficial for individual breast cancer patients in intensifying the value of established proliferation markers (Heidebrecht *et al*, 1997; Rudolph *et al*, 1999, 2001). Prognostication of breast cancer is at the moment based on Ki-67 immunohistochemistry, which has been proven to have strong prognostic correlations (Bouzubar *et al*, 1989; Veronese *et al*, 1993). Our paper suggests that the application of securin alone or in combination with other proliferation markers could contribute to the prognostication of invasive breast cancer.

The observed average level of securin immunopositivity in our present results differs from that of our previous study (Talvinen *et al*, 2008). The obvious explanations lie in the different patient selection procedure and histological material. The present patient data comprise mammographic screening cases characterised by early cancer detection, modern diagnostic and therapeutic methods, and long-term follow-up. Moreover, the type of histological material is an important feature in the observed securin expression. At present, TMAs are also established for staining for cell proliferation (Hoos *et al*, 2001; García *et al*, 2003; Couvelard *et al*, 2009). However, some publications address the possible influence of field selection of TMAs on the observed immunopositivity, especially with regard to uneven staining and expression patterns (Gillett *et al*, 2000; Wärnberg *et al*, 2008; Couvelard *et al*, 2009). This is of special interest with regard to the expression of proliferation markers, which is, according to many investigators, concentrated in ‘hot spots’, that is, the most cellular and invasive front at the periphery of the tumour (Jannink *et al*, 1995; Beliën *et al*, 1999, Salminen *et al*, 2005). Thus, TMA punches do not necessarily represent the most proliferative tumour area and, therefore, the highest securin and Ki-67 expression of the tumour might not be evaluated in this study. This does hamper comparison between reports but does not, however, influence the prognostic conclusions based on a single study with consistent histological and immunohistochemical methods.

In summary, this paper introduces securin as a prognostic factor of breast cancer-specific survival, independent of patient's age, nodal status and tumour size. In combination with Ki-67 immunohistochemistry, securin further intensified the prognostic value of cell proliferation. Although the cut-off point may not directly be applicable to clinical material in whole sections, our results on TMA suggest that minimal or absent securin immunopositivity could indicate a more favourable outcome of disease than that concluded from Ki-67 immunopositivity alone. Securin is a recently published oncogene with multiple functions in tumourigenesis and progression, invasion, and metastasis of malignant disease, and, therefore, further investigations are needed to evaluate the possible prognostic and therapeutic applications of securin in treatment decisions of individual breast cancer patients.

## Figures and Tables

**Table 1:** Clinicopathological characteristics of patient material (*n*=310)

*Age at diagnosis (years)*	
Mean (min to max)	58 (49–78)
	
*Tumour diameter (cm)*	
Mean (s.d.)	2.3 (1.4)
	
*Follow-up time (years)*	
Mean	10.5
(Min to max)	(0.2–19.9)
	
*Tumour size*	
Small (⩽3 cm)	74% (*n*=229)
Large (>3 cm)	15% (*n*=48)
	
*Histological type*	
Ductal	81% (*n*=252)
Lobular	11% (*n*=33)
Other types	7% (*n*=22)
	
*Axillary nodal status*	
Node −	46% (*n*=142)
Node +	45% (*n*=141)
	
*Ki-67 status*	
Low (<10%)	42% (*n*=130)
High (⩾10%)	49% (*n*=153)
	
*Causes of death during follow-up*	
Breast cancer	18% (*n*=55)
Other	20% (*n*=61)

**Table 2:** Summary of mean fractions (%) and standard deviations (in parentheses) of securin and Ki-67 immunopositive nuclei

	**Securin**	**Ki-67**
*All*	6.1 (6.8)	15.0 (14.2)
Ki-67, <10%	3.3 (4.1)	4.5 (2.6)
Ki-67, ⩾10%	8.6 (7.3)	24.0 (14.0)
		
*IDC*	6.4 (6.9)	16.0 (14.8)
Ki-67, <10%	3.5 (4.3)	4.8 (2.5)
Ki-67, ⩾10%	8.5 (7.3)	24.5 (14.5)

The results are based on all patients with invasive breast carcinoma (*n*=310) and on subgroups divided according to invasive ductal histology (IDC) and Ki-67 immunopositivity (low, <10%; high, ⩾10%).

**Table 3:** Summary of Cox's univariate analyses on securin immunopositivity performed in invasive breast carcinomas and in subgroups divided according to invasive ductal histology (IDC) and Ki-67 immunopositivity (low, <10%; high, ⩾10%)

	***n***	***P***	**HR**	**95% CI**
*All*	310	0.006	2.9	1.3–6.0
Ki-67<10%	130	0.057		
Ki-67⩾10%	153	0.523		
				
*IDC*	252	0.014	2.9	1.2–6.8
Ki-67<10%	101	0.064		
Ki-67⩾10%	134	0.626		

The results are based on the optimal cut-point determined at 1.5% of securin immunopositivity, derived from analysis of breast cancer-specific survival of the patient data.

**Table 4:** Summary of multivariate Cox's regression analyses of securin immunoexpression performed in invasive breast carcinomas and in subgroups divided according to invasive ductal histology (IDC) and Ki-67 immunopositivity (low, <10%; high, ⩾10%)

	***n***	***P***	**HR**	**95% CI**
*All*	257	0.028	2.3	1.1–5.0
Ki-67<10%	106	0.031	5.1	1.2–22.7
Ki-67⩾10%	134	0.617		
				
*IDC*	211	0.051		
Ki-67<10%	84	0.024	13.1	1.4–121.3
Ki-67⩾10%	118	0.719		

The results are based on the optimal cut-point determined at 1.5% of securin immunopositivity, derived from analysis of breast cancer-specific survival of the patient data. Axillary lymph node status, patient's age and tumour size at the time of diagnosis were used as covariates in multivariate analyses.
